# The novel circFKBP8/miR-432-5p/E2F7 cascade functions as a regulatory network in breast cancer

**DOI:** 10.1186/s41065-024-00331-1

**Published:** 2024-08-27

**Authors:** Zhongkui Jin, Wang Xu, Kunlin Yu, Cailu Luo, Xiaodan Luo, Tao Lian, Changshan Liu

**Affiliations:** Department of Breast Surgery, Yichun People’s Hospital & The Affiliated Yichun Hospital of Nanchang University, No.1061 Jinxiu avenue, Yiyang New District 336000, Yichun, Jiangxi China

**Keywords:** circFKBP8, miR-432-5p, E2F7, Breast cancer

## Abstract

**Background:**

Circular RNAs (circRNAs) are capable of affecting breast cancer (BC) development. However, the role and underneath mechanism of circFKBP8 (also known as hsa_circ_0000915) in BC remain largely unknown.

**Methods:**

Expression analyses were performed using quantitative real-time polymerase chain reaction (qRT-PCR), western blot, and immunohistochemistry (IHC) assays. Effects on cell functional phenotypes were determined by assessing cell proliferation, migratory capacity, invasion, and stemness in vitro. The relationship between microRNA (miR)-432-5p and circFKBP8 or E2F transcription factor 7 (E2F7) was examined by RNA pull-down, dual-luciferase reporter, and RNA immunoprecipitation (RIP) assays. Xenograft assays were used to identify the function of circFKBP8 in vivo.

**Results:**

CircFKBP8 was presented at high levels in BC tissues and cells. High circFKBP8 expression was associated with worse overall survival in BC patients. CircFKBP8 suppression inhibited BC cell proliferation, migratory capacity, invasion and stemness in vitro. CircFKBP8 suppression blocked xenograft tumor growth in vivo. Mechanistically, circFKBP8 functioned as a miR-432-5p sponge to modulate E2F7 expression. CircFKBP8 modulated BC cell malignant behaviors by miR-432-5p, and miR-432-5p affected these cell phenotypes through E2F7.

**Conclusion:**

Our observations prove that circFKBP8 promotes BC malignant phenotypes through the miR-432-5p/E2F7 cascade, offering a promising therapeutic and prognostic target for BC.

**Supplementary Information:**

The online version contains supplementary material available at 10.1186/s41065-024-00331-1.

## Highlights

CircFKBP8 and E2F7 are notably upregulated, and miR-432-5p is downregulated in breast cancer and cells.

CircFKBP8 suppression inhibits proliferation, migration, invasion, and stemness of breast cancer cells.

CircFKBP8 regulates breast cancer progression through the miR-432-5p/E2F7 axis.

CircFKBP8 accelerates tumor growth in vivo.

**Supplementary Information:**

The online version contains supplementary material available at 10.1186/s41065-024-00331-1.

## Introduction

Breast cancer (BC) is a serious malignant tumor with significant incidence and mortality among women worldwide [1, 2]. With the deterioration of lifestyle and ecological environment, the percentage of new cases of BC in China is as high as 3-4% annually [3]. Therefore, elucidating the pathogenesis of BC, controlling the incidence of BC and improving the therapeutic effect of BC have become major problems to be solved.

Unlike linear RNA, circular RNAs (circRNAs) have a closed-loop structure and are a special class of non-coding RNA, so they are conserved and tissue-specific and not easily degraded by nucleic acid exonucleases [4, 5]. Studies have shown that circRNAs are involved in the occurrence and development processes of various tumors by acting as transcription regulators or sponges of microRNAs (miRNAs) [6]. For example, circSEC24A was upregulated in pancreatic cancer (PC), and increased circSEC24A expression promoted PC progression by upregulating TGFBR2 via miR-606 [7]. Circ_0001367 was reduced in glioma, and its upregulation could enhance LUZP1 expression to inhibit glioma progression by sponging miR-545-3p [8]. Hsa_circ_0008434 was capable of promoting gastric cancer growth and metastasis via the miR-6838-5p/USP9X cascade [9]. Many studies have demonstrated that circRNAs possess critical functions in BC progression. For instance, wan et al. found that circTFF1 regulated BC progression through sponging miR-338-3p and promoting FGFR1 expression [10]. Zhong et al. revealed that circRASSF2 accelerated BC cell malignant behaviors through regulating the miR-1205/HOXA1 cascade [11]. Circ_0084927 was enriched in BC and facilitated BC progression via sponging miR-142-3p to modulate ERC1 expression [12].

According to a previous study, we found that many circRNAs were dysregulated in BC, of which circFKBP8 (also called hsa_circ_0000915 based on the circBase ID), a relatively unexplored circRNA in cancer biology, was significantly upregulated in BC [13], implying the implication of circFKBP8 in BC progression. Furthermore, the function and underlying mechanism of circFKBP8 in BC are unclear. Therefore, we sought to explore the precise action of circFKBP8 in BC.

## Materials and methods

### Tissue sample

70 pairs of BC tissue specimens and adjacent non-cancer tissue specimens were obtained from BC cases with the written informed consent at Yichun People’s Hospital & The Affiliated Yichun Hospital of Nanchang University. All the patients enrolled in this study had not anti-cancer therapy before operation, and their clinicopathological features were presented in Table 1. The Ethics Committee of Yichun People’s Hospital & The Affiliated Yichun Hospital of Nanchang University approved this study with approval No.20,230,218.

**Table 1:** The clinicopathologic features in breast cancer patients

Parameters	*N* = 70
Age, years	
< 50	33
≥ 50	37
Tumor size	
< 2	29
≥ 2	41
TNM stage	
I-II	55
III	15
Lymph node metastasis	
No	52
Yes	18

### Cells and cell culture

Four BC cells (MDA-MB-231, MDA-MB-453, MDA-MB-468, and BT549) and human normal breast cells MCF-10 A were obtained from ATCC (Manassas, VA, USA). All cells were incubated in DMEM medium (Gibco, Carlsbad, CA, USA) with 10% FBS and 1% penicillin/streptomycin. Cells were cultured in a 37℃, 5% CO_2_ incubator with humidified air.

### Cell transfection

For transient transfection, miR-432-5p mimic or inhibitor (miR-432-5p or inh-miR-432-5p), E2F transcription factor 7 (E2F7) overexpressed plasmid pcDNA-E2F7 (E2F7) and their negative controls (RiboBio, Guangzhou, China) were transfected into BC cells using lipofectamine 3000 reagent (Invitrogen). The transfection efficiency was detected 24 h after transfection. For stable knockdown of circFKBP8, three short hairpin RNAs (shRNAs) targeting circFKBP8 (sh-circFKBP8#1: 5’-ACAGCCCGTTCCCTGCCTTGG-3’, #2: 5’-CTCAACAGCCCGTTCCCTGCC-3’, and #3: 5’-CCCGTTCCCTGCCTTGGGGGC-3’) were constructed by RiboBio and transfected into BC cells, respectively. Viral supernatants were obtained and used to infect BC cells for 48 h. To establish stable knockdown cell line of circFKBP8, virus-positive BC cells were selected by puromycin for 2 weeks.

### Quantitative real-time polymerase chain reaction (qRT-PCR)

Total RNA was isolated in Trizol reagent (Invitrogen). The concentration of RNA was determined by NanoDrop2000 (Thermo Fisher Scientific, Waltham, MA, USA). Total RNA was converted into cDNA under use of PrimeScript RT reagent kit (Exiqon, Aarhus, Denmark) with random primers and miRNA-specific primers (miR-432-5p: 5’-CTCAACTGGTGTCGTGGAGTCGGCAATTCAGTTGAGCCACCCAA-3’, miR-490-3p: 5’-CTCAACTGGTGTCGTGGAGTCGGCAATTCAGTTGAGCCACCCAA-3’). The qPCR analysis was performed by SYBR Green I (Takara, Dalian, China) and specific primers. The relative RNA expression was measured by the 2^−∆∆Ct^ method. Primers were shown in Table 2. GAPDH (for circRNA and mRNA) and U6 (for miRNA) were used as the housekeeping genes.

**Table 2:** Primers sequences used for PCR

Name		Primers for PCR (5’-3’)
circ_0000915(circFKBP8)	Forward	CCAAGGCAGGGAACGGG
	Reverse	CGTCTGCAGATGTACGGTGA
E2F7	Forward	GATCGATCAAGGATGGCCCC
	Reverse	TTCCGCTTGCTGTCTGTCAA
miR-432-5p	Forward	GTATGATCTTGGAGTAGGTCA
	Reverse	CTCAACTGGTGTCGTGGAG
miR-490-3p	Forward	GTATGACAACCTGGAGGACT
	Reverse	CTCAACTGGTGTCGTGGAG
GAPDH	Forward	GGAGCGAGATCCCTCCAAAAT
	Reverse	GGCTGTTGTCATACTTCTCATGG
U6	Forward	CTCGCTTCGGCAGCACA
	Reverse	AACGCTTCACGAATTTGCGT

### RNase R treatment

Total RNA was obtained from BC cells. 2.5 µg of RNA was cultured with 4 U of RNase R (20 U/µL, BioVision, Milpitas, CA, USA) at 37℃ for 0.5 h. Then, circFKBP8 and GAPDH levels were detected by qRT-PCR.

### CircRNA localization assay

Total RNA was isolated from the nucleus and cytoplasm of BC cells using PARIS™ Kit (Invitrogen), according to the manufacturer’s instructions. Then, circFKBP8 amount was analyzed via qRT-PCR. U6 and GAPDH were used as nuclear and cytoplasmic controls, respectively.

### CCK8 assay

2 × 10^3^ BC cells were cultured in 96-well plates for 48 h. After that, 10 µL CCK8 reagent was added into cells for additional 2 h. Then, the absorbance (450 nm) value was detected using a microplate reader (Thermo Fisher Scientific).

### 5-Ethynyl-29-deoxyuridine (EDU) assay

BeyoClick™ EdU-647 kit (Beyotime, shanghai, china) was used to detect cell proliferation ability. In brief, BC cells grown in 96-well plates were incubated with 25 µM EDU solution. After 4 h, cells were treated with paraformaldehyde and Triton X-100 (Beyotime). Subsequently, cells were incubated with 100 µL EdU detection solution for 30 min, and cell nuclei were stained with 300 µL diamidine phenylindole (DAPI; Beyotime) for 20 min. Then, pictures were taken under the fluorescence microscope and the EDU-stained cells were scored relative to total cells.

### Colony formation assay

Approximately 500 BC cells were seeded into 6-well plates and incubated for 2 weeks. Then, cells were fastened with methanol (Sigma-Aldrich) for 10 min and stained with crystal violet (Sigma-Aldrich) for 15 min. The number of colonies was counted by Image J software (NIH, Bethesda, MD, USA).

### Transwell assay

BC cells in serum-free DMEM medium were plated into the upper transwell chamber (Corning Inc., Corning, NY, USA) precoated with (invasion assay) or without (migration assay) matrigel (Corning Inc.), and lower chamber was added with DMEM medium plus 20% FBS. After 24 h, the migrated and invaded cells were fixed and stained by 0.1% crystal violet (Sigma-Aldrich). Pictures of cells were photographed under a microscope.

### Sphere formation assay

BC cells were seeded into Ultra-low attachment 6-well plates (Corning Inc.) with DMEM medium, which included epidermal growth factor (100 ng/ml), basic fibroblast growth factor (10 ng/mL), B27 (2%), and Insulin (4 ng/mL) (Sigma-Aldrich). After ten days of culture, pictures of cells were obtained with a microscope.

### Western blot

Total protein was acquired in lysis of radioimmunoprecipitation assay (RIPA) buffer (Sigma-Aldrich). 40 µg protein was loaded for sodium dodecyl sulfate polyacrylamide gel electrophoresis (SDS-PAGE). The resulting gels were transferred to polyvinylidene fluoride membranes (Sigma-Aldrich), followed by blocking in 5% non-fat milk (Invitrogen). The primary antibodies were as follows: anti-E2F7 (1:1,000, AV37583, Sigma-Aldrich, St. Louis, MO, USA), anti-KI-67 (1:1,000, ab92742, Abcam, Cambridge, MA, USA), anti-MMP2 (1:1,000, ab86607, Abcam), anti-Nanog (1:1,000, ab190250, Abcam), and anti-β-actin (1:4,000, ab8226, Abcam). The secondary antibody (1:5,000, ab205718, Abcam) was incubated at room temperature for 1 h. The protein signals were visualized by ECL Kit (Solarbio).

### Dual-luciferase reporter assay

The segments of circFKBP8 or E2F7 3’UTR covering wild-type (WT) and mutant-type (MUT) binding sites of miR-432-5p were constructed into pmirGLO vector (Promega, Madison, WI, USA). Luciferase reporter vectors and miR-432-5p mimic or miR-NC mimic were co-transfected into BC cells through lipofectamine 3000 reagent. 48 h later, luciferase activities were analyzed.

### RNA pull-down assay

C-1 magnetic beads (Life Technologies, Carlsbad, CA, USA) were pre-treated with oligo probe or circFKBP8 probe (Ribobio) for 2 h. Total extractions of MDA-MB-231 and MDA-MB-468 cells were incubated with the probe-bead complex at 4℃ overnight. Then, miR-490-3p and miR-432-5p levels were detected using qRT-PCR.

### RNA immunoprecipitation (RIP) assay

RIP assay was conducted by the RIP Kit (Geneseed, guangzhou, China). In brief, BC cell lysates were mixed with the magnetic beads labeled with anti-Ago2 (ab156780, Abcam) or anti-IgG (ab109489, Abcam) antibody for 12 h. After beads were digested by proteinase K, the enrichment levels of miR-432-5p and circFKBP8 were evaluated by qRT-PCR.

### Tumor xenografts assay

Stable MDA-MB-231 cells (5 × 10^6^ cells/ 0.2 mL PBS) with knockdown of circFKBP8 (sh-circFKBP8#1) or shRNA negative control group (sh-NC) were injected subcutaneously into BALB/c nude mice (6-week-old, *n* = 5/group). Tumor volume was measured every 7 days using the formula: volume= (length×width^2^)/2. After 35 days, tumors were isolated from the sacrificed mice and weighted. The excised tumors were also used to detect the protein levels of E2F7 (ab245655, Abcam), KI-67 (ab15580, Abcam), MMP2 (ab97779, Abcam), and Nanog (ab109250, Abcam) by Immunohistochemistry (IHC). All experiments in nude mice were approved by Animal Ethical Committee of The Ethics Committee of Yichun People’s Hospital & The Affiliated Yichun Hospital of Nanchang University approved this study with approval No.20,230,218.

### Statistical analysis

The results were expressed as mean ± standard deviation (SD). All assays were independently performed with three times. Graphpad Prism 8.0 software was used to analyze data. Two group differences were analyzed by Student’s *t*-test or Wilcoxon signed-rank test. The multiple groups were compared by one-way analysis of variance with Kruskal-Wallis tests. *P* < 0.05 was defined as statistical significance.

## Results

### CircFKBP8 was significantly upregulated in BC tissues and cells

**Fig. 1:**
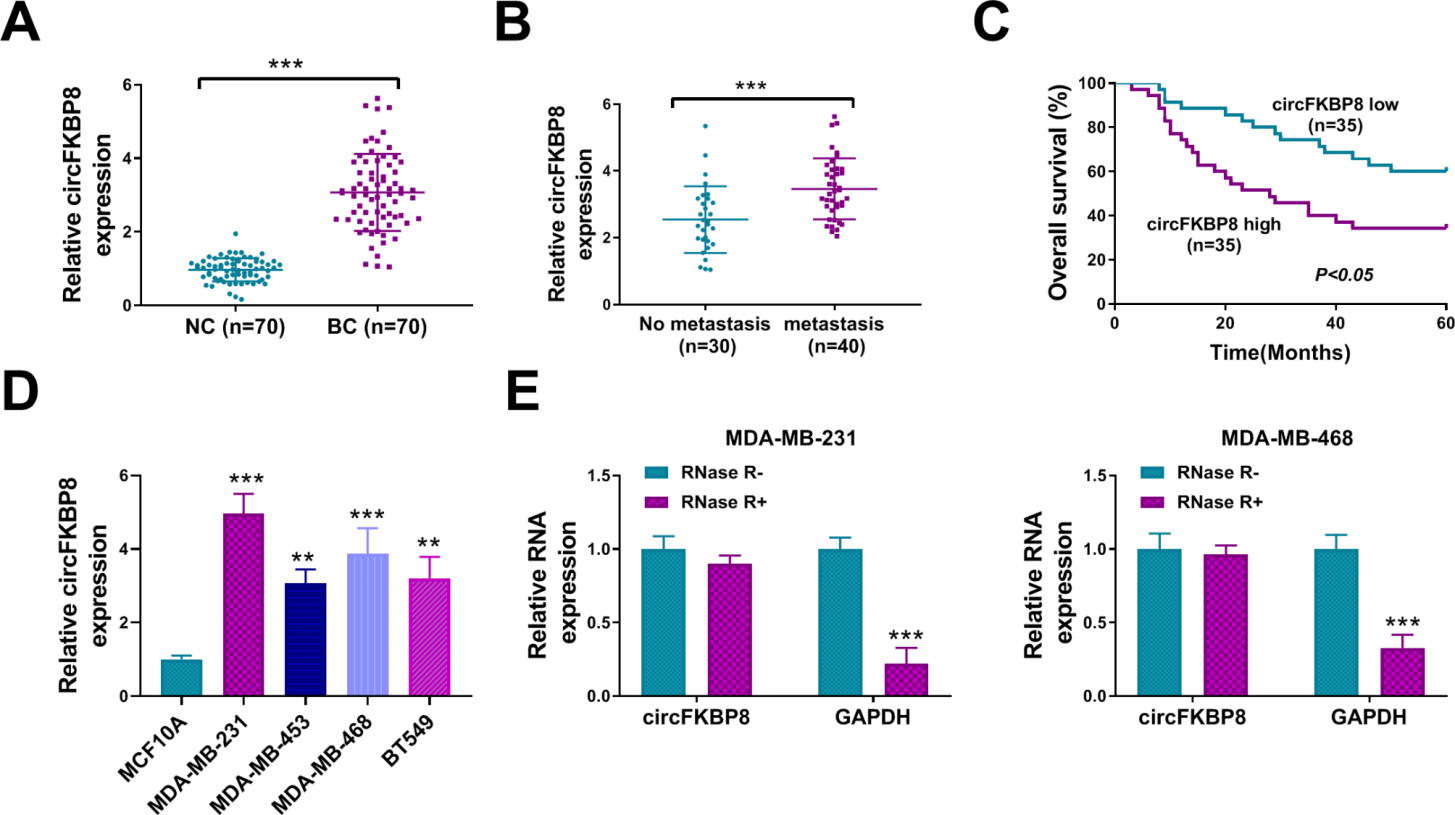
CircFKBP8 was highly expressed in BC. (**A**) CircFKBP8 expression was detected by qRT-PCR in BC tissues (*n* = 70) and adjacent non-cancer tissues (NC) (*n* = 70); (**B**) circFKBP8 expression in BC patients with no metastasis and distant metastasis; (**C**) The prognostic value of circFKBP8 by survival curve analysis; (**D**) circFKBP8 expression in BC cells (MDA-MB-231, MDA-MB-453, MDA-MB-468, and BT549) and human normal breast cells MCF-10 A; (**E**) The expression of circFKBP8 and GAPDH was detected after treatment with or without RNase R in BC cells. ** *P* < 0.01, *** *P* < 0.001

First, we found that circFKBP8 expression was significantly upregulated in BC tissues compared with adjacent non-cancer tissues (Fig. 1A), and circFKBP8 expression was notably increased in BC patients with distant metastasis compared with no metastasis (Fig. 1B). Survival analysis presented that high circFKBP8 expression in BC patients was significantly associated with a poor overall survival (OS) (Fig. 1C). CircFKBP8 expression was strikingly increased in MDA-MB-231, MDA-MB-453, BT549 and MDA-MB-468 BC cells compared to normal MCF10A cells (Fig. 1D). Because of the more significant upregulation of circFKBP8 in MDA-MB-231 and MDA-MB-468 BC cells (*P* < 0.001), we used the two cell lines in the subsequent experiments. RNase R treatment assay demonstrated that circFKBP8 was more stable than the linear GAPDH mRNA when treatment with RNase R (Fig. 1E).

### Knockdown of circFKBP8 inhibited BC cell proliferation, migration, invasion, and stemness

**Fig. 2:**
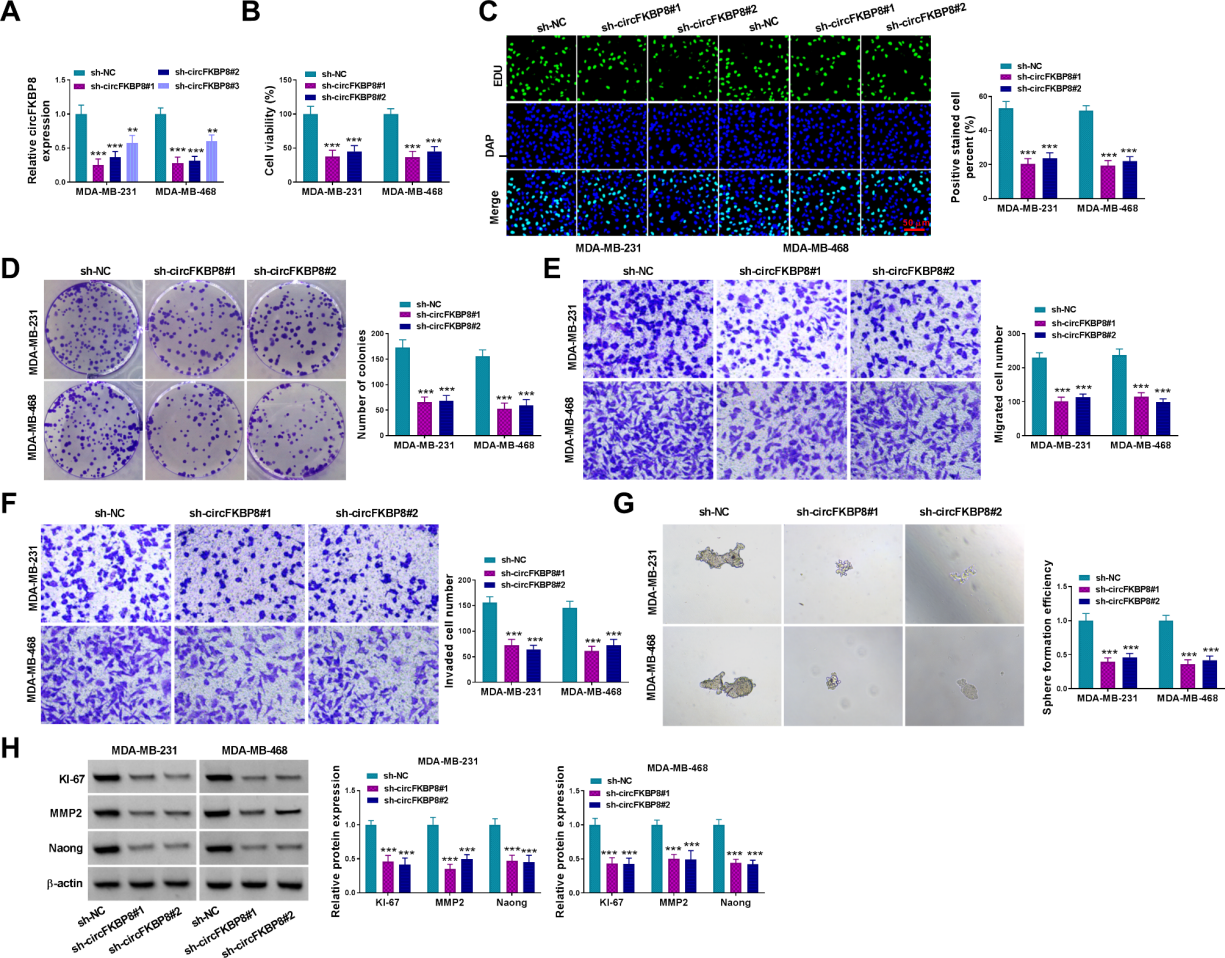
CircFKBP8 inhibition suppressed BC cell proliferation, migration, invasion, and stemness. (**B**) The expression of circFKBP8 was detected after transfected with sh-NC, sh-circFKBP8#1, sh-circFKBP8#2, and sh-circFKBP8#3 in BC cells; (**B-D**) CCK-8 assay, EDU assay and colony formation assay were used to detect cell proliferation; (**E-F**) Transwell migration and invasion assays were performed to assess cell migration and invasion ability; (**G**) Sphere formation assay was used to evaluate cell stemness; (**H**) The protein levels of KI-67, MMP2 and Nanog in BC cells were detected by western blot assays. ** *P* < 0.01, *** *P* < 0.001

To explore the unknown functions of circFKBP8 in BC, we constructed circFKBP8 knockdown cells using three shRNAs targeting circFKBP8 (sh-circFKBP8#1, #2, and #3). According to the qRT-PCR results, three shRNAs could significantly reduce circFKBP8 expression, and sh-circFKBP8#1 had the highest knockdown efficiency (Fig. 2A). CCK8 assay showed that cell viability was inhibited in sh-circFKBP8#1 and sh-circFKBP8#2 groups relative to the sh-NC group (Fig. 2B). EDU and colony formation assays were used to detect BC cell proliferation. The results demonstrated that circFKBP8 knockdown reduced the percent of EdU-positive cells (Fig. 2C) and the number of generated colonies (Fig. 2D) in MDA-MB-231 and MDA-MB-468 cells. We also performed transwell assay, and found that inhibition of circFKBP8 greatly suppressed cell migration and invasion abilities (Fig. 2E-F). In addition, sphere formation assay was used to detect BC cell stemness. The results showed that depletion of circFKBP8 repressed MDA-MB-231 and MDA-MB-468 cell stemness ability (Fig. 2G). We also detected the protein levels of proliferation-related factor KI-67, invasion-related MMP2, and stemness-related protein Nanogin BC cells, and found that silencing of circFKBP8 significantly reduced the expression of KI-67, MMP2 and Nanog proteins (Fig. 2H). These data revealed that circFKBP8 knockdown inhibited BC cell malignant behaviors in vitro.

### CircFKBP8 targeted mir-432-5p in BC

**Fig. 3:**
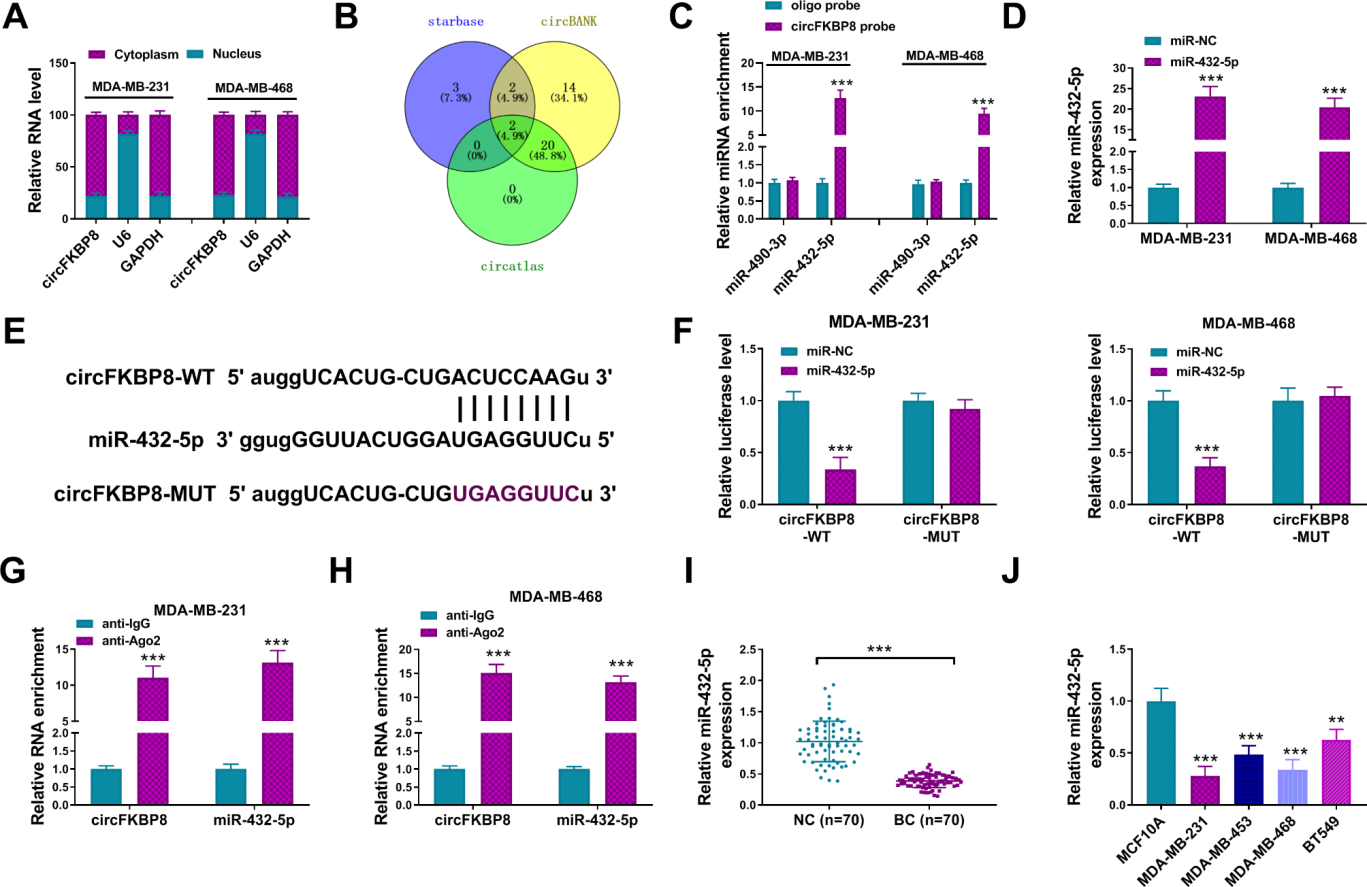
CircFKBP8 bound to miR-432-5p in BC. (**A**) The nuclear and cytoplasmic fractions of circFKBP8 were detected by qRT-PCR in BC cells; (**B**) Prediction of downstream target miRNAs for circFKBP8 binding by bioinformatics websites (starbase, circBank, and circatlas); (**C**) The enrichment of miR-490-3p or miR-432-5p was detected by qRT-PCR after pull-down assay with oligo probe or circFKBP8 probe; (**D**) The expression of miR-432-5p was assessed after transfection with miR-432-5p or miR-NC. (**E**) Schematic diagram of binding sites between miR-432-5p and circFKBP8; (**F**) The luciferase activity was detected using dual-luciferase reporter assay after co-transfection with miR-NC or miR-432-5p and circFKBP8-WT or circFKBP8-MUT; (**G-H**) The enrichment of miR-432-5p and circFKBP8 was evaluated after immunoprecipitation with Ago2 in RIP assay; (**I-J**) miR-432-5p expression was detected in BC tissues and cells. ** *P* < 0.01, *** *P* < 0.001

To explore the underneath mechanism of circFKBP8, we analyzed the location of circFKBP8 in BC cells. As shown in Fig. 3A, circFKBP8 was mainly located in the cytoplasm of MDA-MB-231 and MDA-MB-468 cells. Increasing evidence suggested that circRNAs located in cytoplasm might function as sponges of miRNAs. The online tools circBank (http://www.circbank.cn/index.html), circatlas (http://www.geneseed.com.cn/page464?article_id=481) and starbase (http://starbase.sysu.edu.cn) were used to predict the target miRNAs of circFKBP8 and found two potential miRNAs (miR-490-3p and miR-432-5p) (Fig. 3B). RNA pull-down assay showed that miR-432-5p, but not miR-490-3p, was pulled down by the circFKBP8 probe (Fig. 3C). Then, miR-432-5p was used for the target research of circFKBP8. The data of qRT-PCR exhibited that miR-432-5p mimic transfection effectively induced overexpression of miR-432-5p compared with miR-NC transfection (Fig. 3D). To further demonstrate the relationship between miR-432-5p and circFKBP8, we performed dual-luciferase and RIP assays. According to the predicted binding site between miR-432-5p and circFKBP8, we constructed circFKBP8 WT and MUT reporter plasmids (Fig. 3E). The luciferase reporter activity showed that circFKBP8 could bind to miR-432-5p by the predicted binding site in MDA-MB-231 and MDA-MB-468 cells (Fig. 3F). The results of RIP assay suggested that the anti-Ago2 antibody could enrich circFKBP8 and miR-432-5p (Fig. 3G-H). Moreover, miR-432-5p expression was significantly reduced in BC tissues and cells (Fig. 3I-J). In addition, sh-circFKBP8#1 transfection dose-dependently elevated the amount of miR-432-5p in MDA-MB-231 and MDA-MB-468 cells (Supplementary Fig. 1). These data suggested that circFKBP8 functioned as a sponge of miR-432-5p in BC cells.

### CircFKBP8 regulated BC cell progression by sponging miR-432-5p

**Fig. 4:**
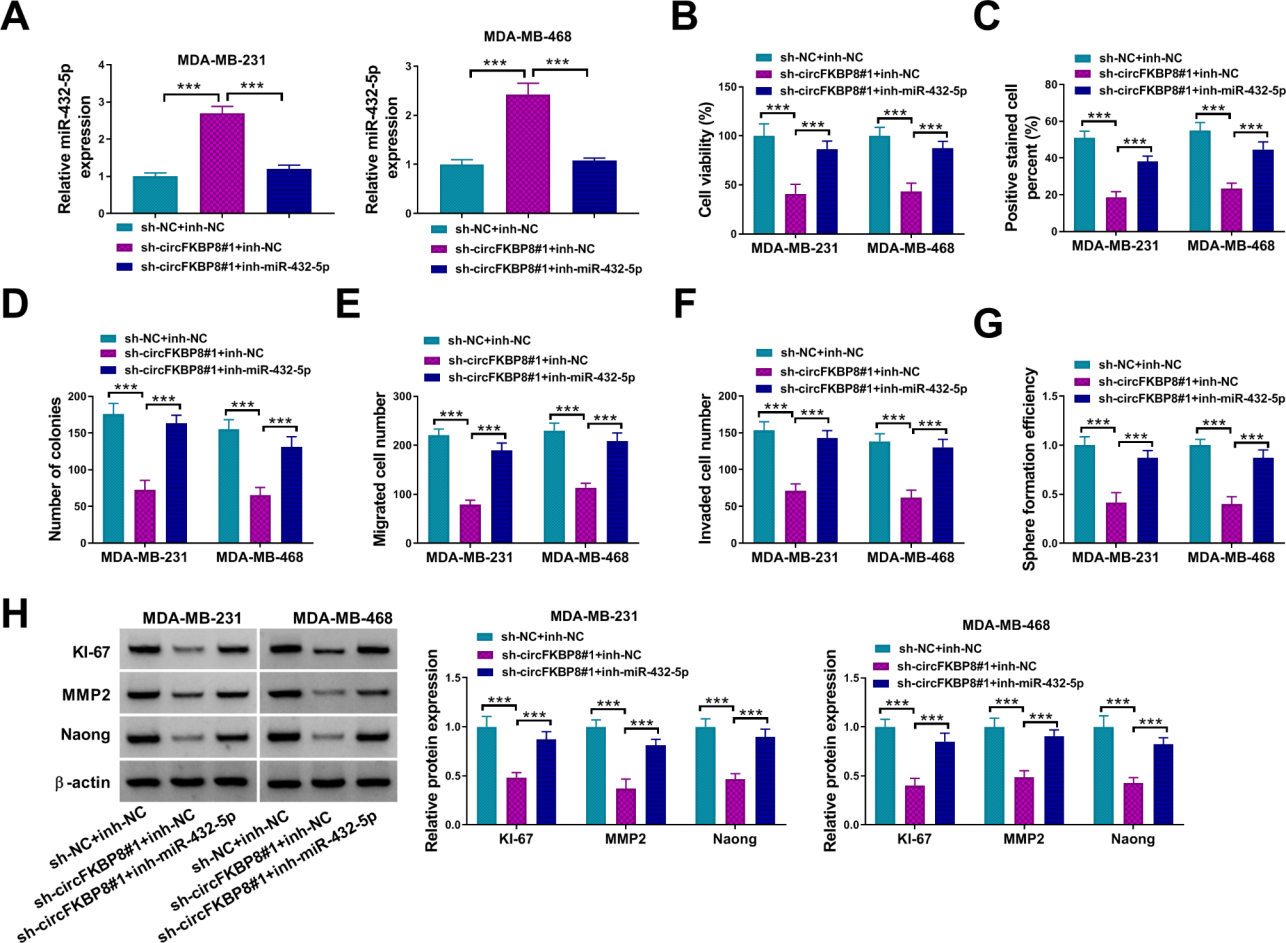
MiR-432-5p inhibition rescued the effect of circFKBP8 knockdown on BC cell malignant behaviors. (**A-H**) MDA-MB-231 and MDA-MB-468 cells were transfected with sh-NC + inh-NC, sh-circFKBP8#1 + inh-NC, or sh-circFKBP8#1 + inh-miR-432-5p; (**A**) miR-432-5p expression was detected by qRT-PCR in MDA-MB-231 and MDA-MB-468 cells; (**B-D**) CCK8 assay, EDU assay and colony formation assay were used to assess cell proliferation; (**E-G**) Transwell assay and tube formation assay were performed to assess cell migration, invasion ability and stemness; (**H**) Western blot assay was performed to assess the protein levels of KI-67, MMP2 and Nanog. ***P < 0.001

To explore the regulatory axis between circFKBP8 and miR-432-5p in BC cells, different plasmids were transfected into BC cells. Silencing of circFKBP8 significantly upregulated miR-432-5p expression, but miR-432-5p expression was partially downregulated by in-miR-432-5p (Fig. 4A). Depletion of miR-432-5p could reverse the inhibition in cell proliferation caused by circFKBP8 knockdown (Fig. 4B-D). Transwell migration and invasion assays showed that circFKBP8 knockdown inhibited cell migration and invasion, but the two impacts were restored by miR-432-5p inhibition (Fig. 4E-F). Suppression of circFKBP8 inhibited cell stemness, while miR-432-5p inhibitor recovered the impact (Fig. 4G). Moreover, circFKBP8 knockdown reduced KI-67, MMP2 and Naong protein levels, whereas miR-432-5p inhibition restored the impacts (Fig. 4H). These data suggested that circFKBP8 regulated BC cell progression by sponging miR-432-5p.

### MiR-432-5p targeted E2F7 in BC

**Fig. 5:**
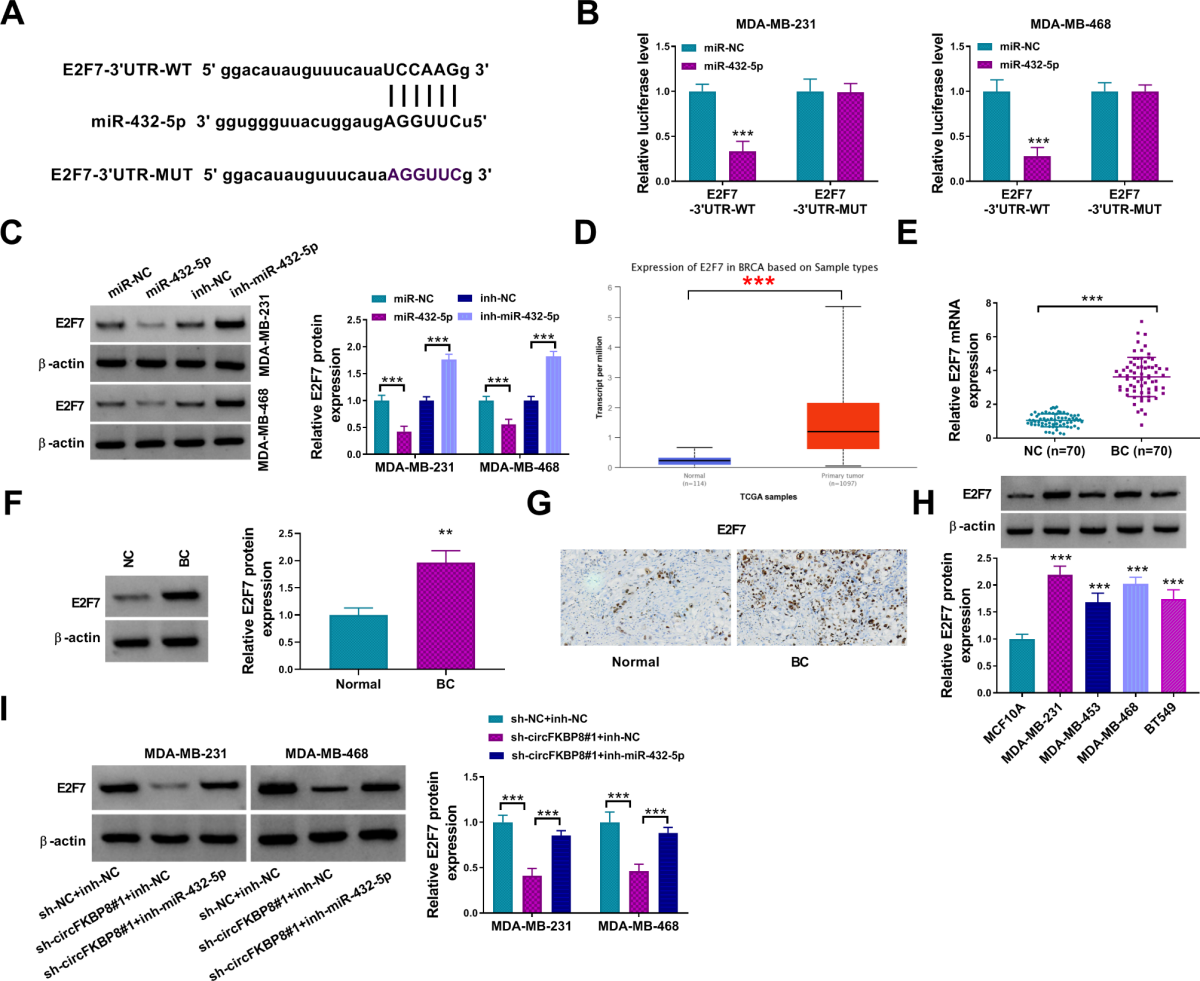
E2F7 was a direct target of miR-432-5p. (**A**) The binding sites was predicted by starbase between miR-432-5p and E2F7; (**B**) Luciferase activity was detected in dual-luciferase assay; (**C**) The protein expression of E2F7 was detected in different groups; (**D**) UALCAN was used to analyze E2F7 expression in BC tissues in TCGA database; (**E-G**) The mRNA (**E**) and protein (**F-G**) levels of E2F7 in BC tissues were analyzed by qRT-PCR and western blot/IHC assays; (**H**) Western blot was used for protein analysis of E2F7 in BC cells; (**I**) After BC cells were transfected with sh-NC + inh-NC, sh-circFKBP8#1 + inh-NC, or sh-circFKBP8#1 + inh-miR-432-5p, the protein level of E2F7 was detected. ** *P* < 0.01, *** *P* < 0.001

Starbase algorithm predicted that E2F7 was a possible downstream target of miR-432-5p (Fig. 5A). Dual-luciferase reporter assays revealed that miR-432-5p could bind to E2F7-3’UTR to target E2F7 (Fig. 5B). Western blot results revealed that miR-432-5p overexpression downregulated E2F7 expression, while miR-432-5p inhibition elevated E2F7 protein level (Fig. 5C). In addition, we analyzed TCGA datasets through UALCAN tool, and found that E2F7 was strikingly increased in BC tissues (*n* = 1097) relative to normal tissues (n-114) (Fig. 5D). Furthermore, E2F7 mRNA (Fig. 5E) and protein (Fig. 5F-G) levels were notably upregulated in BC tissues compared to normal controls. Also, the protein expression of E2F7 was higher in BC cells than that in normal MCF10A cells (Fig. 5H). Moreover, circFKBP8 suppression decreased E2F7 protein expression, while co-transfection with miR-432-5p inhibitor partially increased E2F7 protein expression (Fig. 5I). These data suggested that miR-432-5p directly targeted E2F7 in BC.

### E2F7 knockdown suppressed BC cell proliferation, migration, invasion, and stemness

**Fig. 6:**
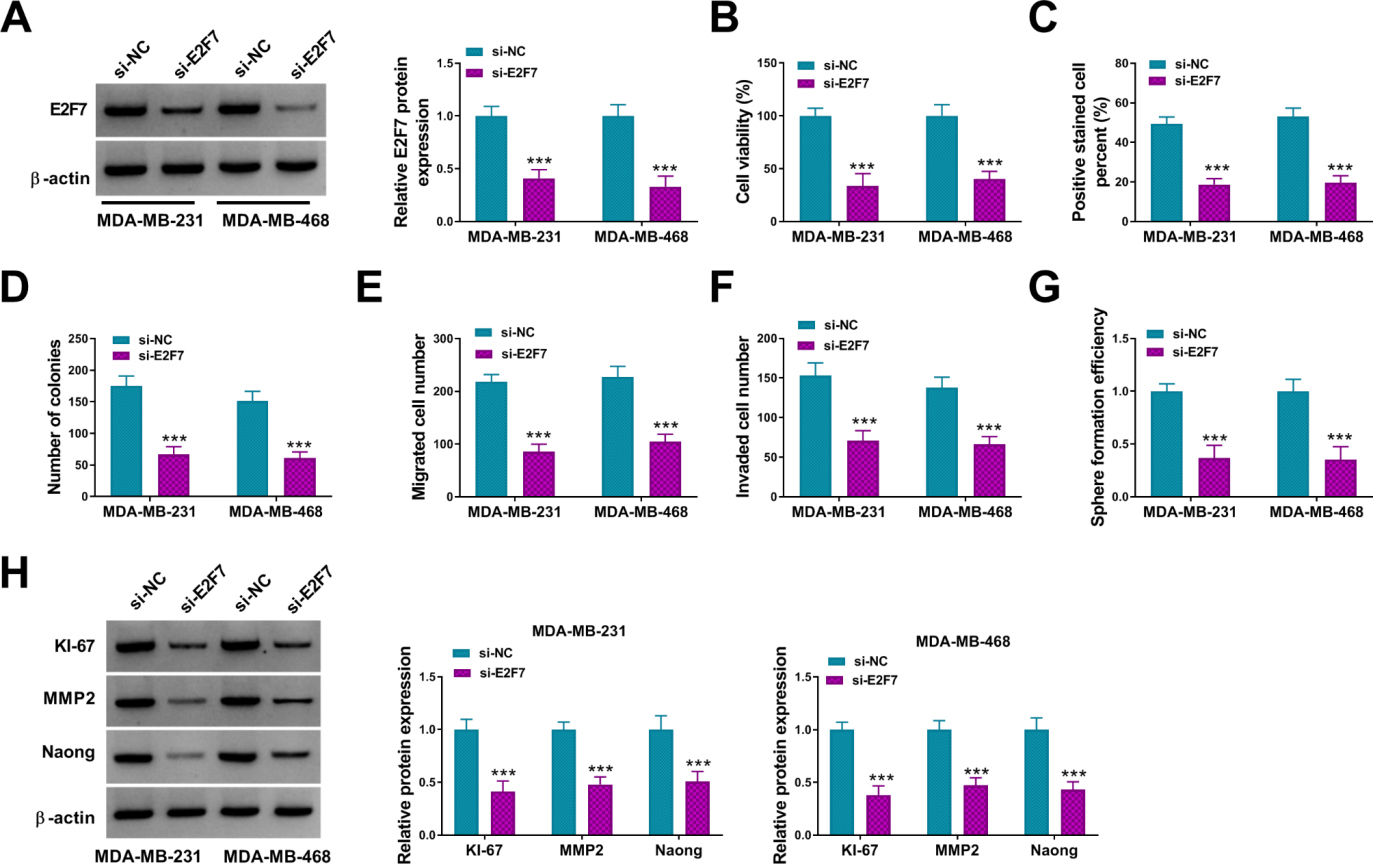
E2F7 promoted BC cell proliferation, migration, invasion, and stemness. MDA-MB-231 and MDA-MB-468 cells were transfected with si-NC or si-E2F7. (**A**) The protein level of E2F7 was detected after knockdown of E2F7 in BC cells; (**B-G**) CCK-8 assay, EDU assay, colony formation assay, transwell migration and invasion assay, and sphere formation assay were used to detect cell proliferation, migration and invasion ability and cell stemness; (**H**) Western blot assay was used to detect the protein levels of KI-67, MMP2 and Nanog in BC cells. *** *P* < 0.001

To further explore the role of E2F7 in BC, the si-E2F7 was used to knock down E2F7. E2F7 protein level was drastically reduced in MDA-MB-231 and MDA-MB-468 cells transfected with si-E2F7 (Fig. 6A). After E2F7 downregulation, the proliferation, migration, invasion and stemness of MDA-MB-231 and MDA-MB-468 cell were evidently inhibited (Fig. 6B-G). In addition, E2F7 inhibition significantly decreased KI-67, MMP2 and Nanog protein levels in MDA-MB-231 and MDA-MB-468 cells (Fig. 6H). These data indicated that E2F7 could promote BC cell malignant behaviors.

### MiR-432-5p suppressed BC cells progression via downregulating E2F7

**Fig. 7:**
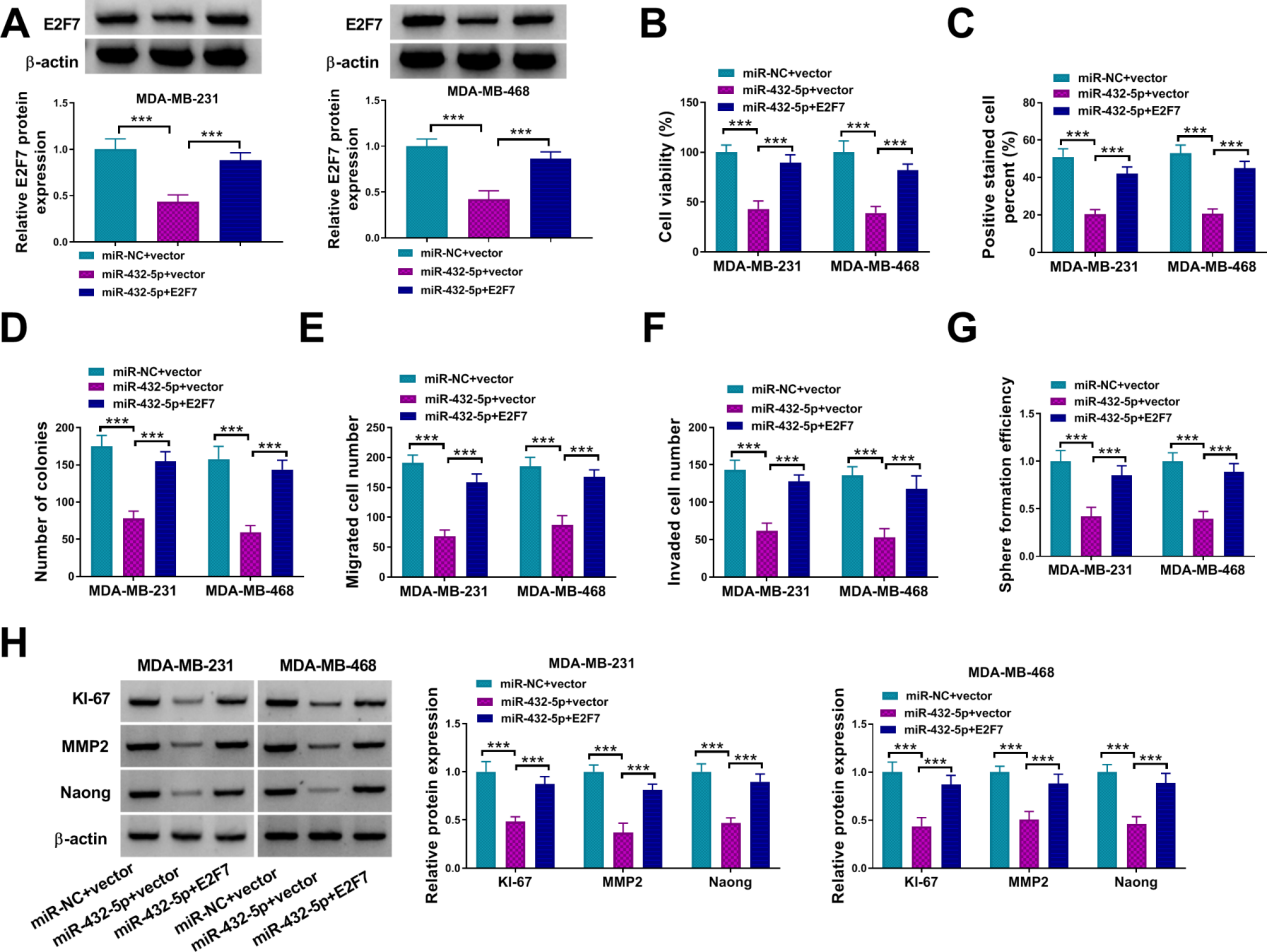
MiR-432-5p inhibited BC cells malignant behaviors by negatively regulating E2F7 expression. (**A-H**) MDA-MB-231 and MDA-MB-468 cells were transfected with miR-NC + vector, miR-432-5p + vector or miR-432-5p + E2F7; (**A**) Western blot analysis was used to evaluate the protein level of E2F7 in BC cells; (**B-F**) The proliferation, migration invasion, and stemness of BC cells were detected by CCK8 assay, EDU assay, colony formation assay, transwell migration and invasion assay, and sphere formation assay, respectively; (**G**) The KI-67, MMP2 and Nanog protein levels were detected in MDA-MB-231 and MDA-MB-468 cells by western blot assays. ***P < 0.001

Considering the relationship between miR-432-5p and E2F7, rescued experiments were performed in BC cells. MiR-432-5p overexpression inhibited E2F7 protein level, while cotransfection of the E2F7 overexpression plasmid partially increased E2F7 protein expression (Fig. 7A). Overexpression of miR-432-5p suppressed the proliferation, migration, invasion and stemness of MDA-MB-231 and MDA-MB-468 cells, which could be reversed by increased E2F7 expression (Fig. 7B-G). Moreover, overexpression of E2F7 restored miR-432-5p mimic-mediated reduction in KI-67, MMP2 and Nanog protein levels (Fig. 7H). These data suggested that miR-432-5p repressed the proliferation, migration, invasion, and stemness by regulating E2F7 expression in BC cells.

### CircFKBP8 promoted BC tumor growth in vivo

**Fig. 8:**
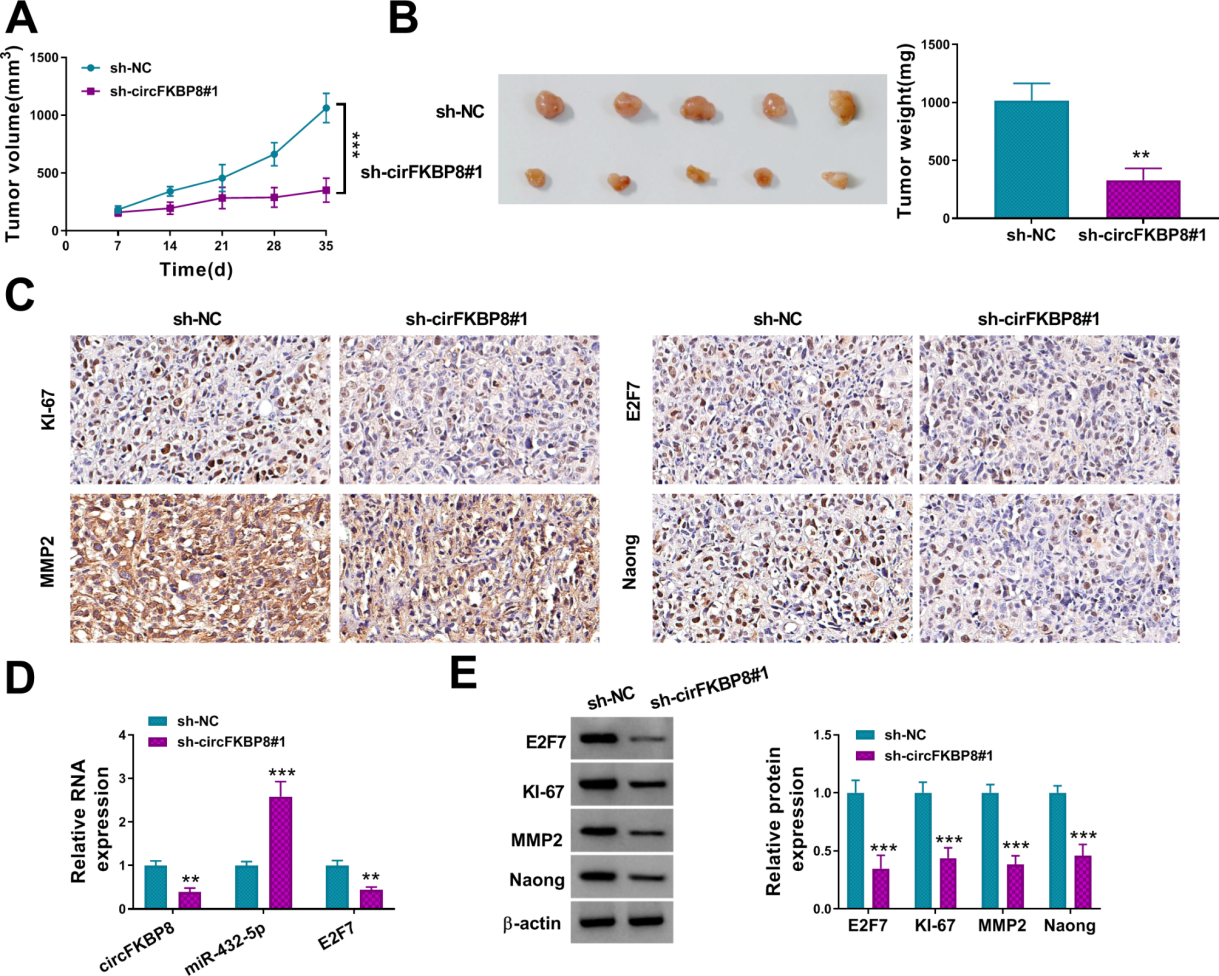
CircFKBP8 knockdown inhibited BC tumor growth in vivo. Xenograft model of sh-NC or sh-circFKBP8#1 group was established in mice. (**A-B**) Tumor volume and Tumor weight were detected in Xenograft assay; (**C**) The levels of KI-67, E2F7, MMP2 and Nanog was assessed by IHC staining in different groups; (**D**) The expression of circFKBP8, miR-432-5p and E2F7 was detected by qRT-PCR in tumors from different groups; (**E**) The protein levels of KI-67, E2F7, MMP2 and Nanog were evaluated by western blot assays in tumors form different groups. ** *P* < 0.01, *** *P* < 0.001

Xenograft assays were used to study the role of circFKBP8 on tumor growth in vivo. After knockdown of circFKBP8, tumor volume and weight were remarkably reduced (Fig. 8A-B). The expression of E2F7, MMP2 and Nanog was decreased in tumor tissues with sh-circFKBP8#1 transduction (Fig. 8C). Moreover, the tumors with sh-circFKBP8#1 transduction had fewer KI-67-positive cells (Fig. 8C). After sh-circFKBP8#1 transduction, the expression of circFKBP8 and E2F7 mRNA was remarkably decreased, but miR-432-5p expression was increased in tumor tissues (Fig. 8D). In addition, circFKBP8 silencing significantly inhibited the protein levels of E2F7, KI-67, MMP2 and Nanog in tumor tissues (Fig. 8E). These data demonstrated that circFKBP8 functioned as an oncogenic circRNA to promote BC tumor growth.

## Discussion

BC is the most prevalent malignancy [14] and the second leading cause of cancer death in women worldwide [15]. Therefore, it is critical to identify promising therapeutic targets for BC treatment.

Increasing evidence suggests that circRNAs are involved in cancer progression [16–19]. For example, circ_0008035 accelerated proliferation and migration of gastric cancer cells [20]. CircCYP24A1 impeded tumorigenesis, cell migration and invasion of renal cell carcinoma [21]. Also, circRNA_002178 knockdown was shown to suppress malignant phenotypes of BC cells [22]. In our study, circFKBP8 was upregulated in BC tissues and cells. CircFKBP8 inhibition suppressed BC cell proliferation, migration, invasion, and stemness. Thus, circFKBP8 could promote malignant behaviors of BC cells in vitro. In addition, silencing of circFKBP8 limited tumor growth in vivo. These results together demonstrated that circFKBP8 acted as an oncogenic factor in BC.

CircRNAs are reported as important regulators in various cancers by acting as sponges of certain miRNAs to mitigate the inhibition of miRNAs on their downstream genes [23–25]. MiR-432-5p repressed colorectal cancer cell growth and invasion through regulating CXCL5 expression [26]. MiR-432-5p inhibited BC cell progression by targeting SLBP [27]. In our study, we demonstrated that miR-432-5p was significantly reduced in BC. MiR-432-5p inhibition restored the suppression effects of circFKBP8 knockdown on BC cell malignant behaviors, indicating that miR-432-5p was a tumor suppressor in BC. The oncogenic function of circFKBP8 may be, at least in part, achieved by miR-432-5p reduction.

E2F7 is a known transcriptional factor and acts as a tumor promoter in various cancer [28, 29]. Overexpression of E2F7 promoted BC cell proliferation [30]. Consistent with the previous study, our data showed that knockdown of E2F7 suppressed cell malignant progression in BC. Furthermore, E2F7 was confirmed as a downstream target of miR-432-5p, and overexpression of E2F7 could rescue anti-tumor activity of miR-432-5p overexpression in BC cells. Thus, E2F7 promoted BC cell progression, and miR-432-5p functioned as a tumor inhibitor in BC via targeting E2F7. The circRNA/miRNA/mRNA axis has been researched in BC progression, such as circ_0103552/miR-515-5p/CYR61 and circ_000554/miR-182/ZFP36 networks [31, 32]. Herein, we found that circFKBP8 downregulation resulted in E2F7 inhibition via releasing miR-432-5p in BC cells. Then, we concluded that circFKBP8 could regulate BC progression via targeting the miR-432-5p/E2F7 axis.

To sum up, our in vitro and in vivo data suggested that circFKBP8 functioned as an oncogenic factor in BC by sponging miR-432-5p to upregulate E2F7 expression. Our findings suggest that circFKBP8 could serve as a novel therapeutic target for BC treatment.

### Electronic supplementary material

Below is the link to the electronic supplementary material.

**Supplementary Material 1:Supplementary Fig. 1** Impact of sh-circFKBP8#1 on miR-432-5p expression in MDA-MB-231 and MDA-MB-468 cells by qRT-PCR. * *P* < 0.05, ** *P* < 0.01, *** *P* < 0.001.

## Data Availability

The analyzed data sets generated during the present study are available from the corresponding author on reasonable request.
