# Supplementary figures and images for: The novel circFKBP8/miR-432-5p/E2F7 cascade functions as a regulatory network in breast cancer
Source: Hereditas. 2024 Aug 27;161:27. doi: 10.1186/s41065-024-00331-1 (PMC11348600; doi:10.1186/s41065-024-00331-1)

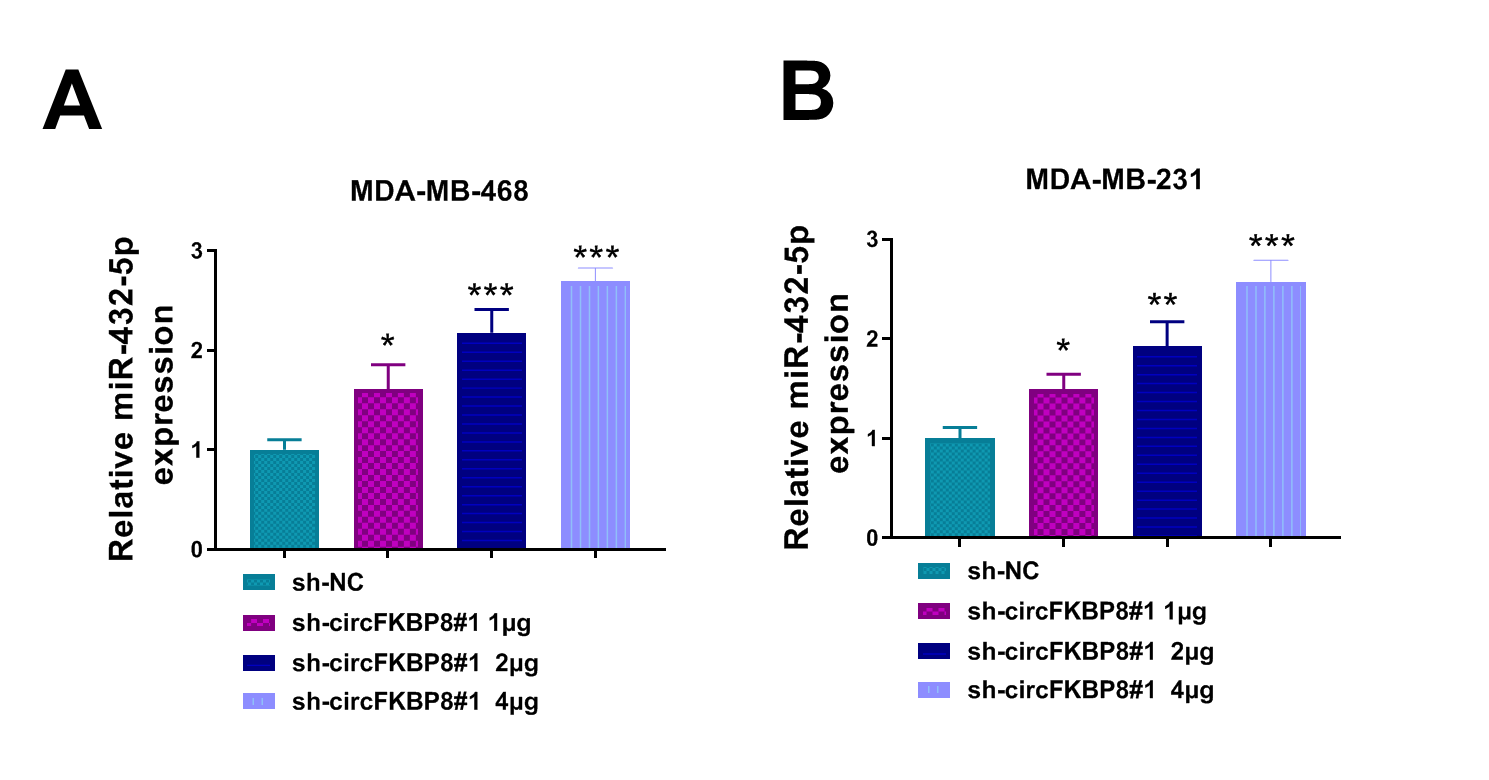

Supplement: Supplementary file 1 — Supplementary Material 1:Supplementary Fig. 1 Impact of sh-circFKBP8#1 on miR-432-5p expression in MDA-MB-231 and MDA-MB-468 cells by qRT-PCR. * P < 0.05, ** P < 0.01, *** P < 0.001. [file 41065_2024_331_MOESM1_ESM.tif]
